# Proteomic Characterization of Inbreeding-Related Cold Sensitivity in *Drosophila melanogaster*

**DOI:** 10.1371/journal.pone.0062680

**Published:** 2013-05-02

**Authors:** Cornelis J. Vermeulen, Kamilla S. Pedersen, Hans C. Beck, Jørgen Petersen, Kristina Kirilova Gagalova, Volker Loeschcke

**Affiliations:** 1 Ecology and Genetics, Department of Biological Sciences, Aarhus University, Aarhus, Denmark; 2 Evolutionary Genetics, Centre for Ecological and Evolutionary Studies, University of Groningen, Groningen, The Netherlands; 3 Department of Genetics and Biotechnology, Faculty of Agricultural Sciences, Aarhus University, Tjele, Denmark; 4 Center for Clinical Proteomics, Department of Clinical Biochemistry and Pharmacology, Odense University Hospital, Odense, Denmark; 5 Department of Biochemistry and Molecular Biology, University of Southern Denmark, Odense, Denmark; 6 Department of Pharmacy and BioTechnology, University of Bologna, Bologna, Italy; University of Massachusetts, United States of America

## Abstract

Inbreeding depression is a widespread phenomenon of central importance to agriculture, medicine, conservation biology and evolutionary biology. Although the population genetic principles of inbreeding depression are well understood, we know little about its functional genomic causes. To provide insight into the molecular interplay between intrinsic stress responses, inbreeding depression and temperature tolerance, we performed a proteomic characterization of a well-defined conditional inbreeding effect in a single line of *Drosophila melanogaster*, which suffers from extreme cold sensitivity and lethality. We identified 48 differentially expressed proteins in a conditional lethal line as compared to two control lines. These proteins were enriched for proteins involved in hexose metabolism, in particular pyruvate metabolism, and many were found to be associated with lipid particles. These processes can be linked to known cold tolerance mechanisms, such as the production of cryoprotectants, membrane remodeling and the build-up of energy reserves. We checked mRNA-expression of seven genes with large differential protein expression. Although protein expression poorly correlated with gene expression, we found a single gene (*CG18067*) that, after cold shock, was upregulated in the conditional lethal line both at the mRNA and protein level. Expression of *CG18067* also increased in control flies after cold shock, and has previously been linked to cold exposure and chill coma recovery time. Many differentially expressed proteins in our study appear to be involved in cold tolerance in non-inbred individuals. This suggest the conditional inbreeding effect to be caused by misregulation of physiological cold tolerance mechanisms.

## Introduction

Virtually all genomes harbor a number of deleterious mutations that, at the population level, contribute to the genetic load. An increase in homozygosity as a result of inbreeding and/or random genetic drift can increase the phenotypic expression of deleterious alleles, and result in the detrimental condition called inbreeding depression [1]. Inbreeding depression can also be caused by the decreased expression of heterozygote superiority and by a breakdown of co-adapted gene complexes due to genetic drift. The exact contribution of these three different genetic causes to the degree of inbreeding depression is largely unknown, but it is generally accepted that recessive deleterious alleles make the largest contribution [2]. Although inbreeding depression is a common and reproducible effect, the underlying genetics are specific to each genetic background and are usually highly complex.

Inbreeding depression is frequently associated with an unusually high sensitivity to environmental change, caused by genotype-by-environment (G×E) interactions acting in a lineage specific manner [3]–[6]. Inbreeding-by-environment (I×E) interactions are a serious threat to long term persistence of populations. It is unclear how, and to what extent, genetic and environmental effects interact in affecting fitness and adaptation in small, isolated populations [7]. This highlights the importance of investigations on environmentally conditioned inbreeding effects.

Inbreeding depression is well understood at the population genetic level, but functional mechanisms are still poorly studied. This is a great omission, as functional genomic studies of inbreeding are invaluable for dissecting many yet unsolved problems within evolutionary biology, conservation biology, agriculture, and medical sciences [7]–[9]. Proteome analysis is suitable as an approach to link genotypic with phenotypic effects at the organismal level, and some studies have been successful in identifying and characterizing genotypic differences using proteomic methods [10]–[13]. With the increasing use of genome-wide technologies, data are emerging that showcase the complexity of the whole organism responses [13]–[16]. Since the data generated by these methods typically are very complex, a powerful strategy is to focus on genetically simple, large and reproducible effects [13], [17]. Similar strategies have been successfully pursued for the study of other complex phenomena, e.g. ageing [18].

The focus of this study is an extreme instance of cold sensitivity in an inbred line of *Drosophila melanogaster* [19]. In most exposed individuals lethality is induced by a combination of mild cold shock and low temperatures. Lethality will not occur without the cold shock. The severity of the effect is affected by dietary conditions [17]. The lethal effect was initially identified in males, but is also expressed in females, which suggests a similar genetic architecture in both sexes. As this inbreeding effect has very specific features, we presumed it to have a relatively simple genetic basis. This inference was supported by a QTL mapping experiment, which implied two major QTL to condition this trait [17].

In this study, an explorative proteomic analysis of the lethal effect is described, where the conditional lethal line was compared with an inbred and an outbred control line. This provided insight into the physiological and molecular processes affected in the conditional lethal line in response to the restrictive conditions, and provided us with candidate genes for future identification at the genotypic level. One question we specifically wanted to address was whether inbreeding depression in a given trait involved disturbances in the physiological mechanisms that underlie this trait, or alternatively involved other unrelated processes. Thus, in particular we wanted to know whether we could trace cold sensitivity in our line to failures in the physiological and biochemical mechanisms of cold resistance. In short, we found a set of 48 differentially expressed proteins in the conditional lethal line, as compared to an inbred and an outbred control line. All proteins are potentially involved in expression of the lethal effect at restrictive conditions. This set was enriched for proteins involved in hexose metabolism, gluconeogenesis and proteins associated with lipid particles. These processes have indeed been associated with cold resistance in previous studies. Finally we used RT-qPCR to check whether differential expression of several selected proteins correlated with transcript abundance.

## Results

### Expression of the Lethal Effect

**Figure 1:**
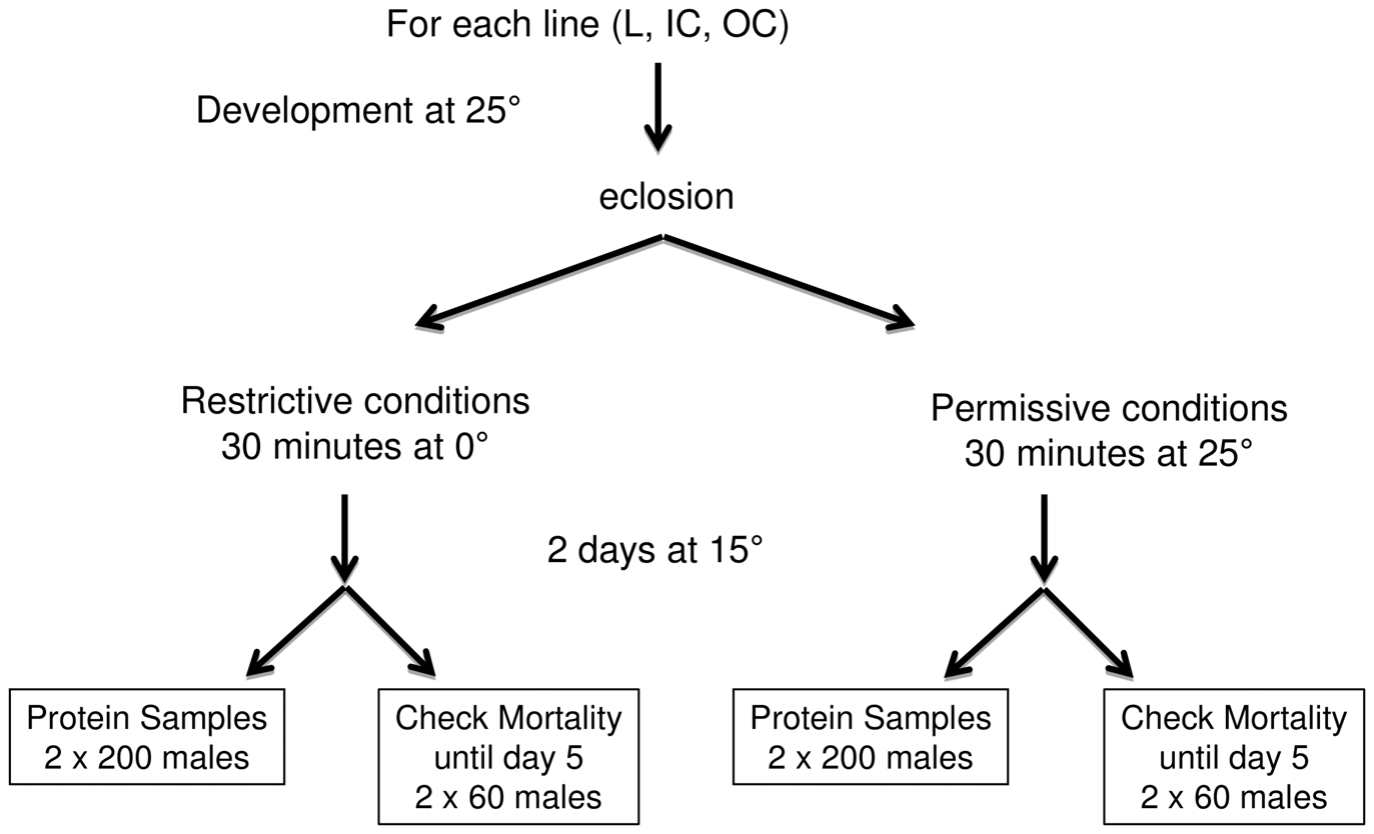
Experimental set-up. Experimental procedure used for obtaining experimental animals. Lines are designated as: OC (Outbred Control line), IC (Inbred Control line) and L (Conditional Lethal line).

**Figure 2:**
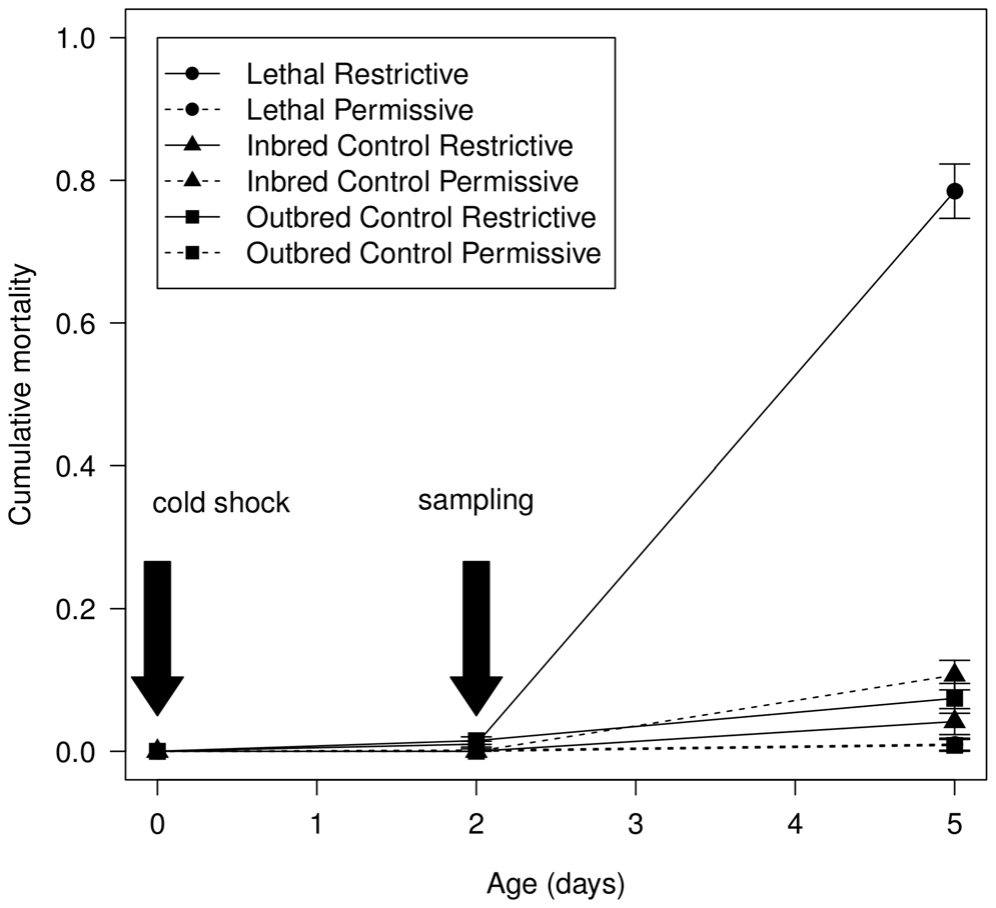
Phenotypic expression of the lethal effect. Cumulative mortality (proportion) for the OC (outbred control) line (▪) and IC (inbred control) line (▴) and the L (conditional lethal) line (•) at the permissive (---) and restrictive temperature (—). Mortality is averaged for the two biological replicates (year 2007 and 2008). Experimental flies received a cold shock shortly after eclosion (day 0). Sampling was performed at day 2, shortly before the onset of the lethal phase (day 3–4).

Male flies from each of the three lines were either cold-shocked at 0°C or kept at 25°C for 30 minutes (Figure 1). After incubation at 15°C for 48 hours, a fraction of the flies from each line was collected and subjected to proteomic analysis. Remaining flies were used to estimate expression of the lethal effect (Figure 2). Mortality in the conditional lethal (L) line at restrictive conditions increased to approximately 80%, contrary to only 1% mortality at the permissive conditions. The outbred control (OC) line and the inbred control (IC) line showed low levels of mortality at both environmental conditions.

### 2-dimensional Gel Electrophoresis (2DGE) and Protein Identification by Mass Spectrometry

**Figure 3:**
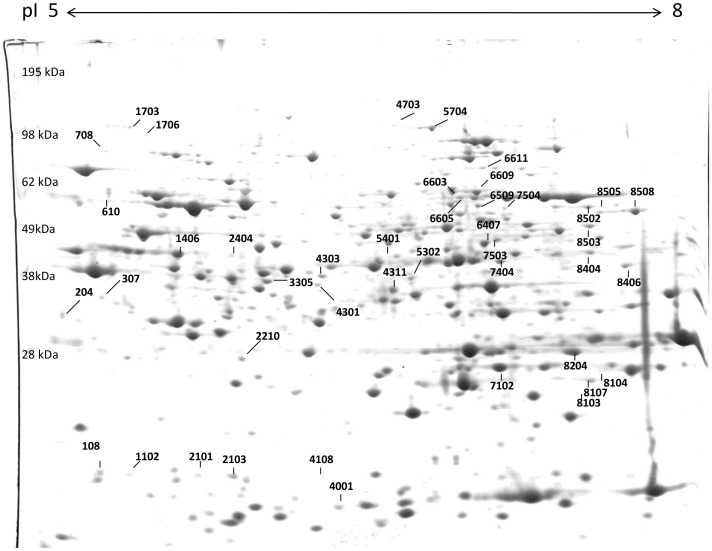
Example 2D gel picture. A representative Coomassie-stained 2D gel showing lysate from flies analysed in the pH 5–8 range. The gel shown is a replicate from the L (conditional lethal) line at restrictive conditions. Numbers indicate differentially regulated protein spots after cold shock. Note the presence of spots SN108, SN610 and SN1102, which are private to this treatment.

More than 1000 proteins were resolved in the pH interval 5–8 (Figure 3). Of these, 44 spots were chosen for identification using mass spectrometry analysis, because they varied significantly (means of triplicates, Student’s *t*-test; *P*<0.05) in one or more contrasts (Table S1). The majority of spots showed a single hit which unambiguously identified the protein. Nevertheless, in some spots several proteins were identified. This can be either caused by co-migrating proteins ending up in the same location on the gel, or because the mass spectrometry analysis was not able to unambiguously identify the protein. Also, some proteins are identified in multiple different spots. For example spots 4703 and 5704 were identified as the same protein, the product of CG1516, isoform E (a putative pyruvate carboxylase). This can be due to different isoforms of the protein migrating differently in the gel, or because of post translational modifications. All proteins are listed in Table 1.

**Table 1:** List of proteins.

Spot number	Protein name	Uniprot accession	Cytological position
108	RH61958p	P29310	46E6–46E8
108	CG18067	A1ZBU8	57A5–57A5
204	CG6206, isoform B	Q8IPB7	31E5–31E5
307	RE08556p	Q8MS44	29F1–29F1
610	beta-Tubulin at 56D, isoform B	Q24560	56D7–56D8
610	angiotensin converting enzyme precursor	Q10714	34E2–34E2
708	glycoprotein 93	Q9VAY2	98B6–98B6
1102	CG18067	See SN108	See SN108
1102	larval serum protein 1 gamma	P11997	61A6–61A6
1406	CG7966	Q9VFZ4	87D10–87D10
1703	CG2918, isoform A	O46067	2F3–2F4
1706	ubiquitin activating enzyme 1	Q8T0L3	46A1–46A1
2101	cuticular protein 49Ab	A1Z8Y3	49A1–49A1
2101	proteasome alpha7 subunit, isoform A	Q9V5C6	46B4–46B4
2103	ubiquitin carboxyl terminal hydrolase	P35122	22D4–22D4
2210	CG11796, isoform A	Q9VPF3	77C3–77C3
2404	CG3534	Q9VEQ0	89E6–89E6
3305	henna, isoform A	P17276	66A12–66A12
4001	dihydropteridine reductase, isoform A	Q9VSU6	67A1–67A1
4001	iron regulatory protein 1B	Q9NFX2	86B4–86B4
4108	cuticular protein 49Ab	See SN2101	See SN2101
4108	Tal	Q9W1G0	60A12–60A12
4301	EG:87B1.3	O46096	2D4–2D4
4303	CG1440, isoform A	Q9W3F6	7E9–7E9
4311	RE37426p	Q5U189	29F5–29F5
4703	CG1516, isoform E	Q7KN97	46B4–46B4
5302	HMG coenzyme A synthase, isoform A	Q7K4Q9	53C1–53C1
5401	CG5384	Q9VKZ8	31D4–31D5
5704	CG1516, isoform E	See SN4703	See SN4703
5704	CG1516, isoform E	See SN4703	See SN4703
6407	Hsp70/Hsp90 organizing protein homolog	Q9VPN5	21B8–21B8
6509	Hsp90-related protein TRAP1	Q7KNF3	42B2–42B2
6509	Hsp90-related protein TRAP1	As above	As above
6601	CG9512	Q9VY05	13A1–13A1
6601	malic enzyme	Q9NIW2	87C6–87C7
6601	Hsp90-related protein TRAP1	See SN6509	See SN6509
6603	pro-phenol oxidase A1	Q27598	54F6–54F6
6605	phosphoenolpyruvate carboxykinase, isoform A	P20007	55D3–55D3
6605	transferrin precursor	O97355	17A9–17A9
6609	pro-phenol oxidase A1	See SN6603	See SN6603
6611	CG7470	Q9VNW6	79A5–79A6
7102	CG6084, isoform A	Q9VTK9	68C15–68D
7404	CG9629	Q8SXQ1	76A3–76A3
7503	CG17259	Q9VQL1	23C5–23C5
7504	Rop	Q07327	64A7–64A7
7504	flare, isoform A	Q9VU68	70A8–70B1
8103	Ecdysone-inducible gene L3	Q95028	65A11–65A11
8104	RH41633p	Q8IGE6	67C4–67C5
8107	CG10863	Q9Y112	64A1–64A1
8204	lethal (1) G0334, isoform D	Q9W4H6	4C14–4C14
8404	CG11594, isoform A	Q9VZJ8	64A4–64A4
8404	CG11594, isoform A	As above	As above
8406	CG3590	Q9VEP6	89E8–89E8
8502	CG10924, isoform A	Q7JXB5	55D1–55D2
8503	phosphogluconate mutase	Q9VUY9	72D8–72D8
8505	CG8036, isoform B	Q9VHN7	85A5–85A5
8508	CG8036, isoform B	See SN8505	See SN8505

List of identified proteins for every spot, together with their Uniprot accession number and the cytological position of the coding gene. Note that some spots contain multiple proteins and that several proteins can be found in multiple spots. See text for details.

### Principal Component Analysis

**Figure 4:**
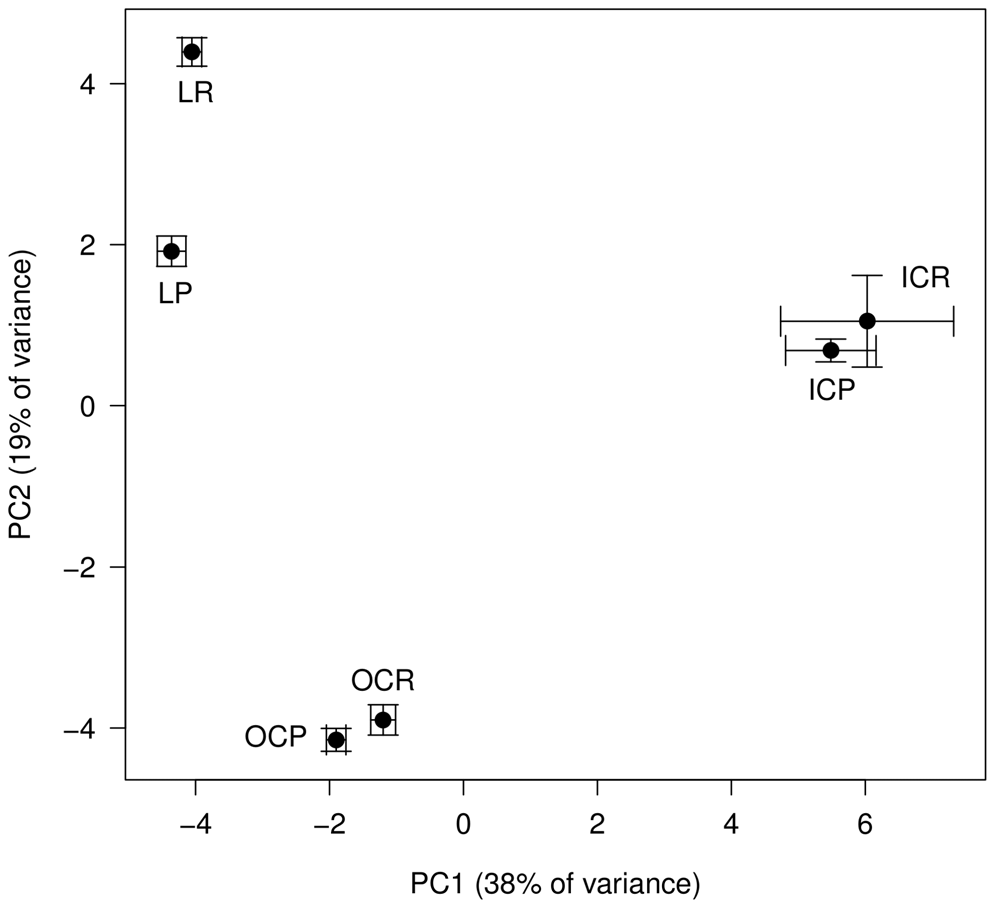
PCA graph of line and treatment. Plot of line and treatment on the two main principal components of protein expression levels. Shown are the positions (means and standard error of means) of all six combinations of line (Inbred Control IC, Conditional lethal L and Outbred Control OC) by treatment (Permissive P and Restrictive R) on a PCA graph of the first and second principal component (PC1, explaining 38% of the variance and PC2, explaining 19% of the variance).

As an exploratory step, we performed principal component analysis (PCA) of the expression data of the 44 selected and 10 control spots. We plotted the six combinations of line and treatment on a PCA-graph of the first two principal components (PCs), which together account for 56% of the total variation in protein expression (Figure 4). PC1 readily separates all lines, but not treatments within lines. This indicates robust line differences in protein levels. PC2 further separates the conditional lethal line at the permissive conditions from the conditional lethal line at the restrictive conditions (LP *vs.* LR in Figure 4). Presumably, the latter pattern is the signature of changes in protein expression levels in response to the expression of the lethal effect. Thus, the PCA confirms that patterns in protein expression capture both constitutive line differences and specific changes associated with the lethal effect.

### Differential Expression

**Figure 5:**
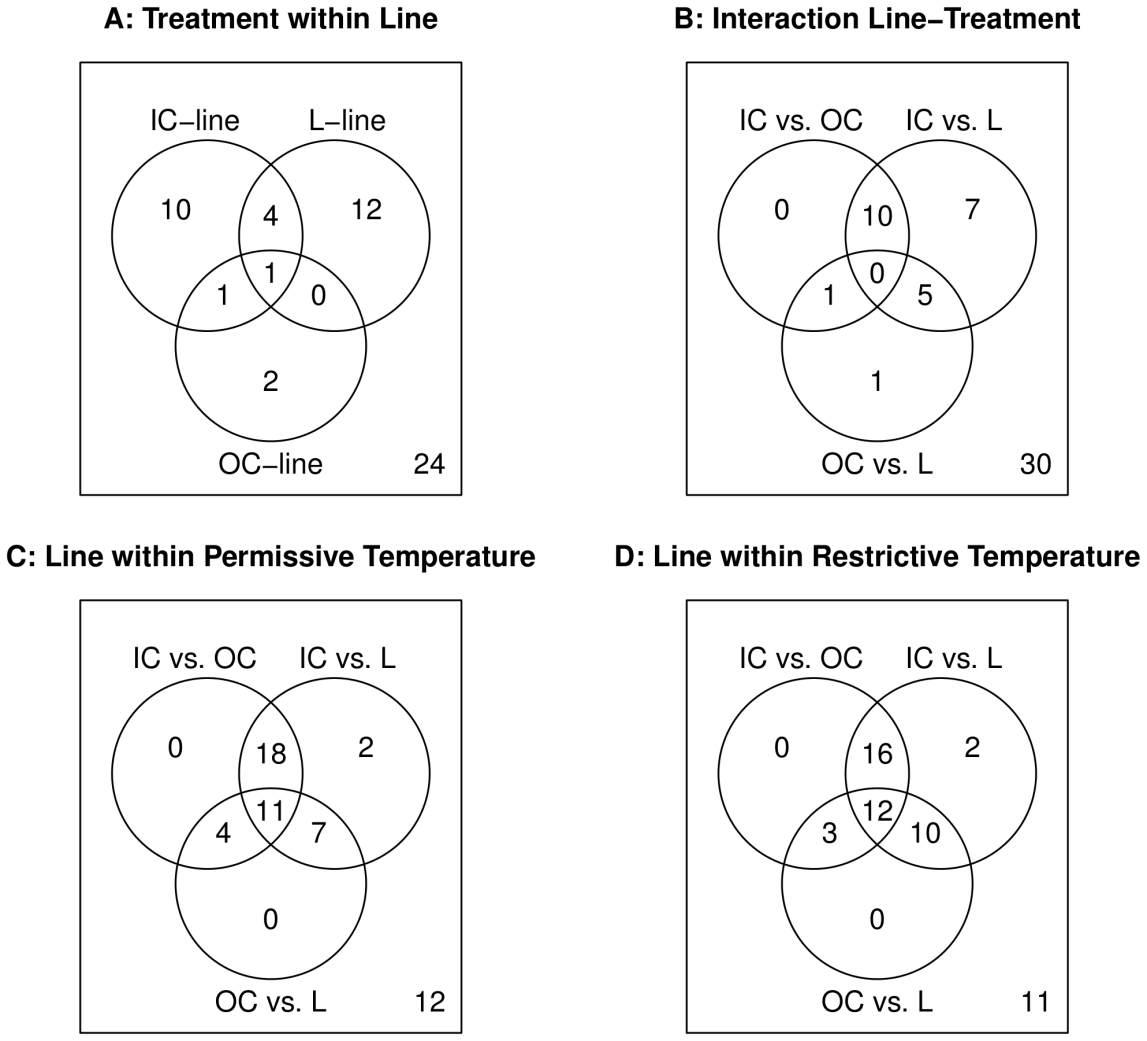
Venn diagrams of the differential expression analysis. A: Venn diagram showing the number of protein spots differentially expressed at the permissive and restrictive temperatures within each line. B: Venn diagram showing the number of protein spots with a significant interaction between treatment and line. C: Venn diagram showing the number of protein spots differentially expressed between lines within the permissive conditions. D: Venn diagram showing the number of protein spots differentially expressed between lines within the restrictive conditions. Lines are designated as: OC (Outbred Control line), IC (Inbred Control line) and L (Conditional Lethal line). The number in the lower right corner indicates the number of nonresponsive spots.

Within lines, 30 spots were differentially expressed between the two treatments (Figure 5a; Table S2). The OC-line was clearly the least responsive line, whereas the IC-line and the L-line had a comparable number of differentially expressed spots. In the L-line, 17 proteins were significantly differentially expressed between the permissive and the restrictive conditions. Out of these 17 proteins, 12 were distinct for the L-line (Figure 5a).

Although expression patterns that are exclusive to the L-line suggest that a spot is associated with the lethal effect, we tried to validate this interaction formally by evaluating line-by-treatment interactions pairwise (Figure 5b, Table S2). We found 24 spots that showed a significant interaction, 13 of which were distinct for the two comparisons involving the L-line (i.e. OC *vs.* L and IC *vs.* L). Expression patterns of some of these spots were spectacular. Three of the differentially expressed spots (number 108, 610 and 1102) were only expressed in the L-line at restrictive conditions, and not at all in the OC-line and the IC-line. Spot 108 and 1102 were both identified as the protein product of *CG18067*, whereas two proteins, beta-Tubulin at 56D and angiotensin converting enzyme precursor were identified for spot 610.

Since we considered it too conservative to only consider spots that show a significant interaction, we also accepted spots with differential expression between lines at either of the environmental conditions (Figure 5c, 5d, Table S2). There are 42 spots with differential expression between lines at the permissive (P) conditions, and 43 at the restrictive (R) conditions. The number of spots that were distinct for the two comparisons involving the L-line was 9 and 12 for the P and R conditions respectively. Again, some of these expression patterns were remarkable. For example, two proteins were expressed at equal levels in the L-line at both environmental conditions (8503: Phosphogluconate mutase and 6603: Pro-phenol oxidase A1), but showed no detectable expression in the OC-line and the IC-line at either condition.

### Candidate Genes

Our previous QTL mapping analysis detected two major QTL at each of the two major autosomes [17]. One QTL (CS2) was localized to the cytological location between 31F and 50F and the second QTL (CS3) was localized to the cytological location between 67B and 71B. The two QTL explained 33% and 39%, respectively, of the phenotypic variance in male mortality during the lethal phase. Seven of the proteins listed in Table 1 are located within the region of CS2, and three in the region of CS3. The genes encoding these proteins (angiotensin converting enzyme precursor, Hsp90-related protein TRAP1, ubiquitin activating enzyme 1, proteasome alpha7 subunit, CG1516, RH61958p and cuticular protein 49Ab in CS2 and RH41633p, CG6084 and flare in CS3) will serve as candidates in follow-up studies.

### GO Enrichment

We tried to find functional categories that were overrepresented in our data set of differential expressed proteins. In order to do so, we matched our protein hits to the appropriate Flybase-gene identifiers [20], [21] and submitted this gene list to the functional annotation and clustering tool of the Database for Annotation, Visualization and Integrated Discovery (DAVID) [22]–[24]. Since we did not possess a custom gene background, we used the default population background, which contains the corresponding genome-wide genes with at least one annotation in the analyzing categories. Functional annotation clustering of our complete gene list returned a cluster of terms, which can be characterized as hexose metabolic process (21 fold enrichment), including the terms gluconeogenesis, glucose metabolic process, pyruvate metabolism and citric acid cycle (Group Enrichment Score = 3.99, corresponding to *P* = 0.0001; Table S3). Another noteworthy and strongly enriched term in the functional annotation was that of lipid particle (7 fold enrichment, *P* = 0.00006).

### RT-qPCR of Selected Transcripts

We have performed RT-qPCR on samples of the L and IC-line to assess whether the absence/presence patterns observed for seven proteins were reflected at the transcript level (Table 2). None of the transcripts show major upregulation in the lethal line at 48 hours after cold shock, which is unlike the pattern in the corresponding proteins. Differential expression was found in *beta-Tubulin at 56D*, *CG18067, prophenol oxidase A1* and *Tal*. The only gene that remotely resembled the pattern of protein expression was *CG18067*, which showed 6-fold upregulation in the L-line after cold shock (LR) with respect to the Inbred Control at permissive conditions (ICP).

**Table 2:** Gene expression of selected proteins.

		Adjusted expression				F-values		
Gene name	Sampling Time	ICP	ICR	LP	LR	Line	Treatment	Line-by-Treatment
Angiotensin converting enzyme	24 hrs	1	1.09	1.09	0.85	0.13	0.12	0.54
beta-Tubulin at 56D	24 hrs	1	1.86	1.08	1.24	0.62	3.46	1.42
CG11796	24 hrs	1	0.88	0.95	1.03	0.11	0.02	0.37
CG18067	24 hrs	1	1.59	1.26	2.54	3.4	9.37**	0.4
phosphoglucose mutase	24 hrs	1	1	1	0.79	0.54	0.54	0.5
prophenol oxidase A1	24 hrs	1	0.79	0.53	0.92	1.75	0.77	4.71*
Tal	24 hrs	1	0.9	1.64	1.01	3.89	3.75	1.55
Angiotensin converting enzyme	48 hrs	1	0.96	1.17	1.12	0.47	0.03	0
beta-Tubulin at 56D	48 hrs	1	3.79	1.61	1.51	0.5	4	4.86*
CG11796	48 hrs	1	0.76	0.77	0.8	0.29	0.35	0.63
CG18067	48 hrs	1	2.06	3.97	6.01	10.29**	2.23	0.17
phosphoglucose mutase	48 hrs	1	1.1	1.64	0.67	0	2.04	3.15
prophenol oxidase A1	48 hrs	1	0.84	1.15	0.1	5.48*	9.36**	7.08*
Tal	48 hrs	1	0.8	2.9	0.33	0.04	8.46*	5.58*

Adjusted mRNA expression values of seven selected proteins at two time points after cold shock. Experimental groups were Inbred Control at Permissive conditions (ICP) and at Restrictive conditions (ICR), and conditional Lethal line at Permissive conditions (LP) and at Restrictive conditions (LR). Expression is relative to ICP. F-values from two-way ANOVA (df = 1,16 for all three factors) are also given. Significant differences are indicated by asterisks: *significant at *P* = 0.05 and **significant at *P* = 0.01.

One of our concerns was that the expression of some genes, especially those restricted to the L-line at restrictive conditions, was a secondary response to the deteriorating condition of the flies of the L-line. We tried to establish whether the genes were differentially regulated in response to cold shock, by assessing gene expression at 24 hours after the cold shock, when flies were still vigorous. The two genes that had differential expression at this time point were *prophenol oxidase A1* and *CG18067*. Also, genes that have differential expression in the inbred control line are likely to be part of a specific response to cold shock. This was found for *CG18067* again, which showed a significant effect of treatment in both lines (*F_1,16_* = 9.37; *P* = 0.007), and also *beta-Tubulin at 56D* (*t_8_* = 2.7; *P* = 0.029), at 48 hours after cold shock.

## Discussion

Since inbreeding depression is typically multifactorial and has different genetic causes across populations, it is often argued that one cannot derive general conclusions from a single instance of inbreeding depression. While this is true, our study is in this respect no different from any other study on inbreeding depression, which also rely on inferences from a single or a few populations. Also, similar considerations have not stopped the study of other complex phenomena. For example, ageing research has greatly benefited from detailed genetic studies of single-gene effects and specific ageing-related diseases [18]. Therefore, we believe that studies like ours are indispensable for progress towards a more detailed understanding of inbreeding depression.

### Proteome Expression Patterns

In this study, we explored the proteome of a cold sensitive conditional lethal line and two control lines in two environmental conditions. Using principal components analysis, we were able to demonstrate robust line differences in protein expression levels, and show interaction with the temperature conditions in the conditional lethal line. This shows that the genetic background is a major determinant of the protein expression profile, but that we can detect environmental disturbances of the proteome as well. Even at permissive conditions, the L-line has a protein expression profile that is clearly distinct from both control lines. The cold shock induces changes of protein expression within the L-line that are smaller in magnitude than the line differences. Constitutive line differences may therefore be as informative to the cause of the conditional lethality as the induced changes.

### Comparison with Other Proteome Studies

We compared our results to those of other proteomic studies of inbreeding depression or cold tolerance. Pedersen *et al.* [13] performed a proteomic analysis of an inbred strain of *D. melanogaster* that suffered from extreme heat-sensitivity. They identified 45 proteins that were differentially regulated in response to the restrictive conditions, with an overrepresentation of proteins associated with oxidative phosphorylation and mitochondria. Five proteins overlapped with the current study (Transferrin 1, CG8036, CG1516, RE08556p/CG9468 and malic enzyme). These proteins may be part of the response to inbreeding, or more generally to cellular stress.

There are no proteomic studies of cold tolerance in *Drosophila* that we are aware of. However, Li and Denlinger [25] performed a proteomic study of rapid cold hardening (RCH) in the brain profiles of flesh flies (*Sarcophaga crassipalpis*). They identified 14 proteins with differential expression between treatments, which were involved in a variety of biological processes, including energy metabolism, protein chaperoning, protein degradation, transcription and cytoskeletal organisation. Although several proteins were identified by their ortholog in *D. melanogaster*, none of these matched those in our list. Although some of our proteins fit a broad category in the Li and Denlinger study (e.g. energy metabolism, protein chaperoning), overall there did not seem to be a convincing overlap. Colinet *et al.* [26] performed a proteomic analysis of cold exposure in the parasitic wasp *Aphidius colemani*. They identified 18 proteins with differential expression between constant and fluctuating cold exposure, which were involved in energy metabolism (glycolysis, citric acid cycle and ATP synthesis), protein chaperoning and protein degradation. Among those proteins, Colinet *et al.* identified the Hsp70/Hsp90 organizing protein homolog, an orthologous protein of the one found in our study (spot 6407). Importantly, six out of 18 proteins were involved in glycolysis or the citric acid cycle. Our set of proteins is also enriched for proteins involved in these biological processes (included in the functional annotation cluster of hexose metabolic process).

A significant portion of the proteins that were differentially expressed in the L-line, as compared to the control lines, were associated with lipid particles. All proteins tagged with this annotation were identified in a proteomic study performed by Beller *et al.* [27]. These are CG1516, henna, CG7470, CG8036, larval serum protein 1 gamma, glycoprotein 93, CG2918 and TRAP1. This suggests that the cold sensitivity of the conditional lethal line is somehow associated with the abundance of lipid particles.

### Correspondence between mRNA and Protein Levels

The striking presence/absence patterns we observed for seven proteins were not reflected at the transcript level. Only *CG18067* echoed this pattern, but showed modest (up to 6-fold) differences in expression. This is consistent with the finding that mRNA and protein profiles often are poorly correlated [28], and suggests that the expression pattern observed at the protein level involved regulation at translation, or posttranslational modification, rather than a massive upregulation of gene expression. Four of the seven genes we investigated showed differential expression (*beta-Tubulin at 56D*, *CG18067*, *prophenol oxidase A1* and *Tal*), demonstrating transcriptional regulation. Gene *CG18067* responds 24 hours after cold shock, and is, together with *beta-Tubulin at 56D*, upregulated after cold shock in the inbred control line. This shows that these genes are regulated in response to cold shock, which supports the idea that the lethal effect involves misregulation of the physiological response to cold shock.

Although expression of transcripts poorly correlates with protein expression, we compared our results to transcriptomic studies of cold tolerance in *D. melanogaster*. Sørensen *et al.* [29] studied differences in whole genome gene expression between lines artificially selected for increased survival during prolonged exposure to cold and their unselected controls. Telonis-Scott *et al.* [30] studied genome wide differential expression between lines artificially selected for reduced chill coma recovery time and their unselected controls. Both studies were included in a reanalysis by Sarup *et al.* [31], which revealed 204 and 307 differentially expressed probe sets in the data from Sørensen *et al.* and Telonis-Scott *et al.* respectively. One probe set from the Sørensen *et al.* study, and three probe sets from the Telonis-Scott *et al.* study correspond to proteins that were differentially expressed in our study (CG11594, isoform A and CG18067, CG7966, CG10924, respectively). Zhang *et al.* (2011) [32] studied transcriptomic responses after multiple and single bouts of cold exposure, and after prolonged exposure to cold in *D. melanogaster*. Four of 730 probe sets that were differentially expressed in their study corresponded to differentially expressed proteins in our study (CG18067, Cuticular protein 49Ab, CG9510 and CG9468). Note that *CG18067*, which responded so strongly at the transcript and protein level in our study, also was differentially expressed in the studies of Telonis-Scott *et al.* and that of Zhang *et al.*

### Quantitative Trait Variation in Cold Tolerance

Studies in *Drosophila melanogaster* have shown that part of the quantitative trait variation for fitness-related traits is due to segregation of rare deleterious alleles maintained by mutation–selection balance [33], [34]. In addition, the physiology underlying some traits may be inherently sensitive to disrupting mutations, resulting in the build-up of genetic load. Therefore, the genetic architecture of inbreeding depression and quantitative trait variation can overlap for some traits.

One of our aims was to detect whether processes disrupted in inbreeding events correspond to those conditioning variation in physiological conditions. Pedersen *et al.* [13] showed that proteins associated with oxidative phosphorylation, mitochondria and muscle function were differentially expressed between an inbred line displaying heat sensitivity and two control lines. In principle, these proteins could inform us about quantitative variation in heat resistance. However, at present there is no known link between oxidative phosphorylation and heat resistance. This does not exclude the possibility that the differentially expressed proteins are responsible for variation in heat resistance, but functional characterisation is needed to resolve this matter.

To assess whether the change in protein expression in the present study concerns cold tolerance mechanisms, we need to briefly summarise the physiology of *D. melanogaster* when confronted with cold stress (reviewed in Doucet *et al.* [35]). (i) Membrane restructuring occurs by changing the phospholipid profiles of cell membranes, a process called homeoviscous adaptation [36]. (ii) In addition, there is an increased production of energy reserves in order to fuel cold-hardening mechanisms, in the form of glycogen, triacylglycerols and proline. (iii) During long-term exposure or recovery there is expression of a diverse set of genes, including protein chaperones. (iv) Metabolic profiling of rapid cold hardening (RCH) and cold shock reveal elevated levels of glucose and trehalose as the most pronounced change after RCH, which are thought to serve as cryoprotectants [37].

In our analysis we detect proteins involved in hexose/glucose metabolic process, in particular in gluconeogenesis (conversion of pyruvate to glucose). These processes fit a pattern of build-up of energy stores and/or the production of cryoprotectants (glucose). Although energy demand is a regular feature of any stressful situation, and has been reported as a general response to inbreeding (e.g. [14]), our case is bolstered by a recent study by Teets *et al.* [38]. They studied the transcriptomic and metabolomic responses during RCH and recovery from cold shock in *Sarcophaga bullata*. Gluconeogenesis and pyruvate metabolism were enriched in the metabolomic and transcriptomic datasets during recovery from cold shock. This was correlated with an upregulation of Phosphoenolpyruvate carboxykinase (PEPCK), which catalyses the rate-limiting step of gluconeogenesis. Remarkably, this protein was also differentially expressed in our data set (SN6605). Another feature of our protein set was that many proteins were associated with lipid particles. Lipid particles are involved in lipid storage and lipid trafficking, and may be involved in membrane remodeling or the build-up of energy stores. The considerable overlap with our previous study [13] suggests that a considerable fraction of the identified proteins are a general indirect response to increased homozygosity, so we cannot rule out that transcriptional and translational activity is increased in adipose tissue as a general stress response. This is a general issue in expression studies of inbreeding depression, as it is difficult to separate primary cause from consequences [8,9, but see 39].

### Mechanism of the Inbreeding-by-environment Interaction

Inbreeding depression becomes, on average, more severe in a stressful environment [4], [40]. However, there exist many exceptions where inbreeding depression does not aggravate during stress, and inbreeding-by-environment (I×E) interactions tend to be stress specific [41], [42]. Reed *et al.* [43] hypothesized that I×E interactions occur because 1) some of the genetic load has environmentally conditional expression, 2) inbred individuals are overall weaker and therefore less capable of coping with stress and 3) stress increases the opportunity for purifying selection, by increasing the amount of phenotypic variation. These hypotheses are not mutually exclusive. The conditional lethal effect in our study is an extreme instance of I×E interaction. It appears to be caused by a misregulation of the cold stress response, and only becomes phenotypically expressed during cold stress. This provides support for hypothesis 1, which depends on conditional expression and also for hypothesis 3, which posits an increase in fitness variance as a result of stress, for example because of the uncovering of cryptic genetic variants. It is more difficult to reconcile our system with hypothesis 2, since the inbred individuals are not overall weaker, but have specific sensitivity to a particular environment. We expect that the addition of more studies of the underlying mechanisms of I×E interactions will show what general patterns, if any, exist.

### Conclusion

Using a proteomic approach, we have explored an extreme case of cold sensitivity in *D. melanogaster*, brought about by inbreeding depression. We found common themes with expression studies of cold tolerance. Our set of differentially expressed proteins was enriched for proteins involved in glycolysis and the citric acid cycle. In another proteomic study of cold resistance, performed in the parasitic wasp *Aphidius colemani* these categories were also overrepresented [26]. Furthermore, we found enrichment for proteins involved in hexose metabolic process (including gluconeogenesis) and lipid particles, which might function in known cold tolerance mechanisms, such as the production of cryoprotectants, membrane remodeling and the build-up of energy reserves [38]. We delineated several candidate genes responsible for the cold sensitivity phenotype in the L-line. The protein product of *CG18067* was restricted to the L-line in restrictive conditions. We showed *CG18067* to be transcriptionally upregulated in response to cold shock, both in the L- line and in the control line. This gene was shown previously to be differentially expressed in lines of *D. melanogaster* artificially selected for reduced chill coma recovery time, as well as wildtype *D. melanogaster* exposed to cold. These facts make this gene an interesting candidate for cold tolerance studies. Compared to the control lines, the L-line has differential expression of proteins associated with the physiological response to cold shock, which suggests that the lethal inbreeding effect is caused by a misregulation of this system.

## Methods

### Stocks

All lines of *Drosophila melanogaster* originate from the G83 base population (see description in [17]) and were started in 1997 [3]. The LI10 conditional lethal line is a highly inbred line (L-line, F∼0.95) that shows a sharp increase in adult mortality at 15°C after cold shock at modified food medium (restrictive conditions), but shows wild type levels of survival at standard laboratory conditions (permissive conditions). The extreme cold sensitivity of the L10 line was discovered in 1999 and expression of the lethal effect at restrictive conditions has been consistently reproducible [44]. The temporal stability of the lethal effect suggests that either the causal alleles are fixed, or strong selection is acting to maintain the alleles in the L-line. The O6 outbred line (OC-line, F∼0.00) and the highly inbred line CI13 (IC-line, F∼0.95) where chosen as control lines. Both lines show wild type levels of survival at both permissive and restrictive conditions. Experimental flies were obtained using a modified protocol as used for a QTL-mapping experiment [17], see Figure 1. Flies from culture were kept in fresh bottles with food and live yeast at 20°C for 3 days. For each of the three lines, four females and one male were collected in each of 40 vials and allowed to oviposit for 2 days at 25°C. Then the food pellet was transferred to plastic bottles to ensure low density and flies were allowed to develop at 25°C. Male flies (0–12 hrs) were collected into glass bottles and either cold-shocked at 0°C or kept at 25°C for 30 minutes. Thereafter flies were transferred to fresh vials. Altogether 32 vials with 20 males were collected for each line and kept at 15°C for 2 days (48 hrs). For each sample, 200 males were snapfrozen in liquid nitrogen and kept thereafter at −80°C. No dead flies were sampled. Remaining flies were used to estimate expression of the lethal effect (Figure 2). The experiment was repeated twice with one year interval. This resulted in 6 line-treatment combinations within each set, in total 12 samples.

### 2-dimensional Gel Electrophoresis (2DGE) and Protein Identification by Mass Spectrometry

Protein samples from the different lines and treatments (6×2) of *D. melanogaster* flies were analysed in triplicates (36 gels in total) by 2DGE 17 cm pH 5–8 linear IPG strips (Bio-Rad Laboratories). Protein samples were re-suspended in 7 M urea, 2 M thiourea, 2% Chaps, 0.002% bromophenol blue, 50 mM dithiothreitol (DTT), 0.2% w/v carrier ampholyte, pH 3–10 (Bio-Rad Laboratories) and used to re-hydrate the IPG strips. Protein load was 800 µg for 17 cm strips. Re-hydration was performed over-night under passive conditions, and iso-electric focusing (IEF) was performed using an Protean IEF cell (Bio-Rad Laboratories) for a total of 65 kWh at 8,000–10,000 V. Prior to loading on the second dimension, focused IPG strips were equilibrated sequentially in a buffer (Tris-HCl buffer containing 6 M urea, 30% v/v glycerol, 2% SDS) containing 2% DTT or 2.5% iodoacetamide for 30 min each and applied to 12% polyacrylamide gels (17 cm). SDS-PAGE was carried out using a Höefer SE 600 system (GE Health Care). Proteins were resolved at a constant voltage of 55 V for 18 hours at 5°C and visualized using a colloidal Coomassie stain [45]. Gels were scanned using an Epson V750 M Pro scanner (Espon, Hemel Heamsteaed, UK) and analysed using the PDQuest 2D software (Bio-Rad Laboratories). Protein spots were excised from the gels, in-gel digested and analysed by LC-MS/MS as described below.

Means of data triplicates from the 2D analysis were subjected to a two-sided Student’s *t*-test at 95% confidence interval in order to designate differentially regulated protein spots (*P<0.05*). Tryptic peptides were analysed by capillary-LC MS using a CapLC XS (Waters) coupled to a Micromass q-TOF II instrument (Micromass, Manchester, UK). The sample (6.4 µl) was loaded onto a home-made 0.5-cm fused silica pre column (150 µm inner diameter, 360 µm outer diameter, packed with C18 (Reprosil Pur C18, Dr. Maish GmbH) using an autosampler. Sequential elution of peptides was done using a linear gradient from solution A (2% acetonitrile in 0.1% formic acid) to 100% solution B (80% acetonitrile in 0.1% formic acid) in 60 min over the pre-column in line with a home-made resolving column (15 cm length×75 µm I.D.), packed with C18 material. The resolving column was connected to a distal coated fused silica emitter (20 µm inner diameter, 340 µm outer diameter, 10 µm tip inner diameter, New Objective, Cambridge, MA). The flow rate was 200 nl/min.

The mass spectrometer was operated in positive ion mode with a resolution of 9,000 at full-width half-maximum using a source temperature of 80°C and a nitrogen counter current flow rate of approximately 60 L/h. MS analyses were performed using 2-s scans. Instrument settings for data-dependent analysis were performed using the three most abundant ions in each cycle MS 2 sec (m/z 300–1500) and maximum 10-sec MS/MS (50–2000), 60 s-dynamic exclusion. Processing of raw data was done using external calibration with fragment ions of glufibronectin resulting in mass errors of typically 10–20 ppm in the m/z range 50–2000. Raw data were processed using ProteinLynx Global Server 2.0 (smooth 4/2 Savitzky Golay, centre four channels/80% centroid). The resulting MS/MS dataset was exported in MicroMass pkl format for automated peptide identification using an in-house MASCOT server (version 2.1.3) (Matrix Sciences, London, UK). Searches were performed against the NCBI non-redundant database – restricted to *Drosophila* proteins, with following search criteria; tryptic peptides, one missed-cleavage allowed; ±50 ppm tolerance for MS and 0.2 Da for MS/MS fragment ions; deamidation of asparagines and glutamine, carbamidomethylation of cysteine, and oxidation of methionine were specified as variable modifications. Six proteins were attributed to other species than *D. melanogaster*. For all of these cases, the entry could be replaced by a closely related ortholog.

### Principal Component Analysis and Analysis of Differential Expression

Prior to statistical analysis raw expression levels quantified from the 2D gels were manually checked. Expression values of zero were taken as missing values in the statistical analysis if the spot was quantified in at least one out of the three replicate gels. If a spot in all three replicate gels was quantified as being absent, or below the detection limit, it was taken as a real-zero expression level, and to ease the transformation, set to 1. Raw expression values were ^2^log transformed before statistical analysis. All data were analyzed using R (version 2.10.0), a programming language and development software for statistical computing and graphics [46].

^2^Log-transformed expression data were subjected to a principal component analysis as implemented by the prcomp function in R. Missing values were imputated by the SVDimpute algorithm as implemented in the “pcaMethods” package [47].

Linear modeling and empirical Bayes methods, implemented in the package Linear Models for Microarray Analysis (LIMMA), were used to assess differential expression of proteins [48], [49]. The linear model included the factors genotype (OC-line, IC-line, L-line), environmental conditions (Permissive and Restrictive) and set (year 2007 and 2008), and all possible interactions. Contrasts for each spot evaluated pairwise differences in expression between lines within the two environmental conditions and a treatment-by-line interaction term using a modified t-test. The number of false positives due to multiple testing was controlled using the Bonferroni method. Differential expression of spots was considered significant if the adjusted *P*-value was below 0.05.

### RT-qPCR of Selected Transcripts

Experimental animals were obtained as described above, but eggs were counted to control density (100 per vial with 9 ml medium). Some samples were collected at an additional time point (24 hrs after cold shock).We collected five samples of five males for each experimental group. RNA was extracted from frozen samples using TRIzol reagent (Invitrogen), using the manufacturer's protocol. Contaminating genomic DNA was digested by incubation with DNAse (DNA-free™ Kit, Applied Biosciences) for 15 minutes at 37°C. Then, RNA was converted to cDNA using RevertAid H minus first strand cDNA synthesis kit (Fermentas), using a 1∶6 mixture of oligo-dT primers and random hexamers.

We developed primers for seven selected transcripts (CG18067, beta-Tubulin at 56D, Angiotensin converting enzyme, CG11796, Phosphogluconate mutase, Pro-phenol oxidase A1 and Tal) and five reference genes (alpha-Tubulin at 84B, forkhead domain 68A, Elongation factor 1alpha100 E, Eukaryotic initiation factor 1A and RNA polymerase II 215 kD subunit) using both PerlPrimer (http://perlprimer.sourceforge.net/) and the NCBI primer design tool (http://www.ncbi.nlm.nih.gov/tools/primer-blast/). We tried to design primer sets that annealed to exon junctions, or that spanned a large intron. This succeeded for all primer sets, except CG18067. All primer sets were checked in silico to confirm that no primer-dimer or secondary structures in the amplicon were formed. We validated all primer sets by PCR on cDNA, and checked for amplification of the correct product size, and absence of non-target amplicons. Also, primer sets were tested on gDNA to confirm that no amplicon was produced from genomic template. We performed RT-qPCR on dilution series to assess the efficiency of the primer sets, and confirm a linear relationship between concentration and Cp-values. Information on all primers can be found in Table S4.

RT-qPCR was performed on an Applied Biosystems 7300 Real Time PCR System, using SYBR Green technology. The temperature profile was: 95°C for 15 min, followed by 45 cycles of 95°C for 15 s, 56°C for 30 s and 72°C for 30 s. The reaction mixture consisted of one microliter cDNA mix (dilution 1∶25) and ABsolute™ QPCR SYBR Green ROX (500 nM) Mix (Abgene, Hamburg, Germany) using a 300 nM primer concentration. Quantification of *Pro-phenol oxidase A1* transcripts required different parameters: cDNA was undiluted, primer concentration was 50 nM and the annealing temperature was set at 58°C. Every run included appropriate non-template controls (NTC) to check for amplification of non-target sequences or contaminations. In addition, a standard ABI 7300 dissociation curve was applied at the end of each run to control for nonspecific amplification.

Data were analysed using the R-package “qpcR” [50]. Raw Rn values were processed by sigmoidal curvefitting as implemented in the function modlist, and initial fluorescence was estimated by the window-of-linearity method as implemented in sliwin [51], [52]. Initial fluorescence values were transformed logarithmically (base 2), to remove dependence of the variance on the mean. Log-transformed initial fluorescence values of the five reference genes were averaged to obtain a mean reference value for each biological sample. The reference value was used to adjust the expression values of the genes of interest. Adjusted fluorescence values were analysed using two-way ANOVA with line, treatment and their interaction as fixed factors.

## Supporting Information

Table S1 **Protein expression levels in the three lines OC-line, IC-line and the L-line at the permissive (Perm.) and restrictive (Restr.) conditions, and the fold change (F.C.) for spots included in the statistical analysis.**
(PDF)

Table S2 **Decision table results from the limma analysis. The table lists for every protein spot, whether a specified contrasts was significant or not.**
(XLS)

Table S3 **Output from functional annotation clustering at the DAVID website.**
(XLS)

Table S4 **Primer sequences used in RT-qPCR experiment, and their associated information.**
(XLS)
