# Supplementary material for: Proteomic Characterization of Inbreeding-Related Cold Sensitivity in Drosophila melanogaster
Source: PLoS One. 2013 May 2;8(5):e62680. doi: 10.1371/journal.pone.0062680 (PMC3642220; doi:10.1371/journal.pone.0062680)
Supplement: Table S1 — Protein expression levels in the three lines OC-line, IC-line and the L-line at the permissive (Perm.) and restrictive (Restr.) conditions, and the fold change (F.C.) for spots included in the statistical analysis. (PDF) [file pone.0062680.s001.pdf]

Supplemental Table 1

Protein expression levels in the three lines OC-line, IC-line and the L-line at the permissive (Perm.) and restrictive (Restr.) conditions, and the fold change (F.C.) for spots included in the statistical analysis. The 10 spots listed under the thin solid line are spots not identified by mass spectrometry, but included because they show a constitutive expression pattern. Significant differential expression between the permissive and the restrictive conditions are indicated with stars; *** : $p < 0.001$, ** : $p < 0.01$, * : $p < 0.05$.

	Outbred-Control line				Inbred-Control line				Cond lethal line			
Spot #	Perm.	Restr.	F.C.		Perm.	Restr.	F.C.		Perm.	Restr.	F.C.	
108	0	0			0	0			0	1226	> 10	***
204	0	0			3072	2288	1.34		1151	1155	1.00	
307	1825	1212	1.51		2360	2063	1.14		1119	379	2.96	***
610	0	0			0	0			0	233	> 10	***
708	3106	3976	1.28		3209	5128	1.60	**	831	860	1.03	
1102	0	0			0	0			0	589	> 10	***
1406	8295	9037	1.09		1022	789	1.30		9900	8482	1.17	
1703	924	996	1.08		1447	2751	1.90	*	926	608	1.52	
1706	1305	1512	1.16		743	923	1.24		1831	1105	1.66	
2101	583	698	1.20		269	404	1.50		311	412	1.32	
2103	1422	1458	1.03		165	311	1.88	***	2415	2038	1.18	
2210	0	0			0	0			1643	972	1.69	***
2404	470	399	1.18		95	109	1.14		609	283	2.15	***
3305	2417	1987	1.22		1468	1851	1.26		3287	2140	1.54	
4001	263	762	2.90	*	2588	1757	1.47	*	1358	1267	1.07	
4108	0	0			0	0			593	216	2.75	*
4301	620	354	1.75		165	214	1.29		1086	441	2.47	**
4303	1092	938	1.16		590	464	1.27	***	1427	1132	1.26	
4311	4052	3391	1.19		321	309	1.04		5169	4269	1.21	
4703	1415	838	1.69		412	417	1.01		454	145	3.14	***
5302	662	411	1.61	*	609	591	1.03		937	698	1.34	
5401	611	469	1.30		0	230	> 10	***	915	2029	2.22	
6407	4417	3856	1.15		3476	3705	1.07		5653	5015	1.13	
6509	1651	1751	1.06		563	835	1.48		1590	1485	1.07	
6601	3499	1272	2.75	***	8974	6855	1.31		2495	1893	1.32	
6603	0	0			0	0			422	393	1.07	
6605	1720	840	2.05	***	137	0	> 10	***	1455	680	2.14	***
6609	939	883	1.06		0	362	> 10	***	4470	3284	1.36	*
6611	1067	855	1.25		230	157	1.46	***	606	371	1.63	
7102	5500	4827	1.14		4793	4462	1.07		18595	12912	1.44	**

7404	4704	3889	1.21	1214	1039	1.17		4522	3843	1.18	
7503	1035	999	1.04	569	340	1.67	***	1293	1167	1.11	
7504	287	246	1.17	0	76	> 10	***	630	361	1.75	**
8103	811	594	1.37	286	292	1.02		1996	1800	1.11	
8104	2904	2274	1.28	857	671	1.28	***	2835	1898	1.49	*
8204	10075	8357	1.21	6445	4612	1.40	**	10494	8626	1.22	
8404	0	0		1917	1569	1.22		2485	1791	1.39	
8406	303	188	1.61	343	0	> 10	***	2396	1948	1.23	
8502	645	642	1.00	731	675	1.08		2007	1565	1.28	
8503	0	0		0	0			2695	2235	1.21	
8508	9865	8435	1.17	3305	1734	1.91	***	10549	6114	1.73	**
5704	3557	2466	1.44	1390	1406	1.01		4481	2195	2.04	*
8505	3467	2730	1.27	2103	1684	1.25		1117	516	2.17	*
8107	3503	3572	1.02	0	0			0	0		
4103	8939	8105	1.10	9811	8568	1.15		9525	7695	1.24	
2201	10862	10052	1.08	10048	8764	1.15		11675	10200	1.14	
1203	4134	5046	1.22	5888	4834	1.22		4708	4450	1.06	
3402	5567	5660	1.02	6981	6822	1.02		5824	4904	1.19	
3405	3742	3448	1.09	3217	3515	1.09		3757	3229	1.16	
2302	4119	4735	1.15	6621	5067	1.31	*	5059	5375	1.06	
4104	3356	3256	1.03	3036	3304	1.09		3503	3473	1.01	
2104	6049	5769	1.05	6666	5759	1.16		6157	5476	1.12	
3104	1927	2171	1.13	1985	1905	1.04		1889	1937	1.03	
3102	1308	1270	1.03	1379	1130	1.22		1316	1173	1.12	
